# Relationship Between Adipose Tissue and Liver Dysfunction in Women with Polycystic Ovary Syndrome and Metabolic Syndrome

**DOI:** 10.3390/metabo16060393

**Published:** 2026-06-05

**Authors:** Sebastião Freitas de Medeiros, Gustavo Arantes Rosa Maciel

**Affiliations:** 1 Department of Gynecology and Obstetrics, Medical School, Federal University of Mato-Grosso, Cuiabá 78060-900, Brazil; 2 Tropical Institute of Reproductive Medicine, Cuiabá 78043-306, Brazil; 3 Discipline of Gynecology, São Paulo University, São Paulo 05403-000, Brazil; garmaciel@gmail.com

**Keywords:** polycystic ovary syndrome, metabolic syndrome, adipokines, hepatokines, hepatic steatosis, hyperandrogenism

## Abstract

Polycystic ovary syndrome (PCOS) is frequently accompanied by visceral obesity, insulin resistance, low-grade chronic inflammation, and metabolic syndrome (MetS). These alterations promote significant dysfunction in adipose tissue and liver metabolism through cytokine production. Growing evidence indicates that the interaction between hepatokines and adipokines plays a central role in the development of metabolic and hepatic abnormalities in women with PCOS. This narrative review was conducted to analyze the relationship between adipose tissue dysfunction and liver metabolic impairment in women with PCOS, emphasizing the involvement of hepatokines and adipokines in insulin resistance, inflammation, hepatic steatosis, hepatic fibrosis and MetS. From this perspective, contemporary clinical, biochemical, and molecular studies were reviewed to evaluate how adipocyte-derived factors and hepatocyte-derived cytokines influence metabolic homeostasis in the liver and adipose tissue in women with PCOS. Increased visceral adiposity in PCOS enhances the release of free fatty acids (FFAs) to the liver, resulting in hepatotoxicity, oxidative stress, and hepatic inflammation. Several hepatokines, including fetuin-A, angiopoietin-like protein 3 (ANGPTL3), selenoprotein P(Sep-P), and hepassocin (HPS), show abnormal circulating levels in PCOS and are strongly associated with insulin resistance, dyslipidemia, and progression to hepatic steatosis. In contrast, fibroblast growth factor 21 (FGF-21), follistatin, and interleukin (IL-6) may exert dual effects. Adipokines, such as resistin, visfatin, apelin, and retinol-binding protein 4 (RBP-4), contribute to chronic inflammation, impaired glucose metabolism, androgen excess, and hepatic steatosis and fibrosis. Some of these adipokines, such as leptin and vaspin, may exert both beneficial and detrimental effects, while others, including chemerin and omentin, appear to play predominantly beneficial roles in metabolism. Reduced adiponectin-to-leptin levels further aggravate metabolic dysfunction. These changes indicate that adipose tissue–liver crosstalk is a key mechanism linking PCOS and MetS. Overall, metabolic disturbances in PCOS are strongly mediated by dysregulated communication between adipose tissue and the liver. Altered hepatokine and adipokine profiles contribute to insulin resistance, liver dysfunction, hypertension and the development of MetS in women with PCOS. Understanding these intricate interactions may support the early identification of high-risk patients and the development of targeted therapeutic strategies.

## 1. Introduction

Metabolic syndrome (MetS) comprises arterial hypertension, visceral obesity, dyslipidemia, hyperuricemia, and dysglycemia associated with insulin resistance (IR) [1]. This condition is frequently found in women with polycystic ovary syndrome (PCOS), in whom the prevalence ranges from 10% to 48% in different populations, depending on the Rotterdam phenotype [2,3,4,5]. To confirm a PCOS diagnosis, clinicians must consider menstrual irregularities, hyperandrogenism, and polycystic ovary morphology (PCOM), while excluding thyroid diseases, hyperprolactinemia, late-onset congenital adrenal hyperplasia, and androgen-secreting adrenal or ovarian tumors [6]. Although PCOS was recently renamed polyendocrine metabolic ovarian syndrome (PMOS), the abbreviation PCOS will be maintained throughout this review without affecting any conclusions. Regarding the etiology of PCOS, evidence suggests that IR, accompanied by compensatory hyperinsulinemia, contributes to increased ovarian androgen secretion [7]. Combined hyperinsulinemia andhyperandrogenemia are strongly associated with visceral adiposity, adipocyte dysfunction, and increased adipose tissue mass, which are closely linked to PCOS and MetS [8,9,10].

Regarding androgens, longitudinal studies evaluating their dynamics are scarce; due to diurnal variation in androgen concentrations, blood samples should be collected in the morning. Taking into account the menstrual criteria for diagnosing PCOS, infrequent menses is defined as a menstrual cycle of ≥45 days or ≤8 menstrual periods per year. Amenorrhea is defined by the absence of menstruation for ≥90 days. Ovarian morphology in PCOS, determined by ultrasound, is defined by a volume ≥10 cm^3^ and/or the presence of ≥12 follicles in at least one ovary [2,3]. Clinical hyperandrogenism, present in about half of patients, is primarily visible by dermatological markers including hirsutism, acne, and androgenetic alopecia. Hirsutism, with a prevalence ranging from 23% to 70%, is characterized by the growth of terminal hair in male-pattern distribution. Acne has been reported in 15% to 46%, resulting from androgen stimulation of the pilosebaceous unit. Androgenetic alopecia, with a prevalence ranging from 5% to 50%, according to ethnicity, is characterized by shortened anagen and progressively thinner hair until the follicle stopsproducing hair.

Several anthropometric parameters are abnormal in PCOS. Overweight or central obesity is present in over 60% of patients [8,9]. Metabolic abnormalities in carbohydrate and lipid metabolism in PCOS are even more frequent [10]. Insulin resistance (IR) is found in 50–70% of women, type 2 diabetes mellitus (T2DM) occurs in about 30%, metabolic dysfunction-associated steatosis liver disease (MASLD) affects approximately 40%, and dyslipidemia, particularly increased triglycerides (TG) and reduced high-density lipoprotein cholesterol (HDL-C), affects up to 70% of patient [9,10].This review focuses on the relationship among obesity, adipocyte-derived biomarkers, and liver dysfunction in PCOS. When possible, it also addresses the association between PCOS and MetS. It is well-established that visceral adipose tissue dysfunction in obese women with PCOS and MetS is linked to significant systemic and hepatic abnormalities [11,12,13,14].

## 2. Crosstalk Between Hepatocytes and Adipocytes

**Figure 1:**
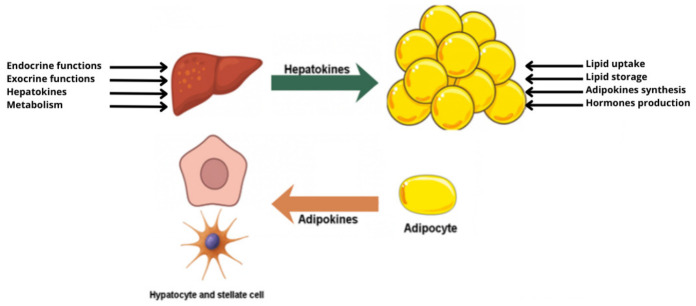
Crosstalk between hepatocytes and adipocytes through selected cytokines.

Many organs and tissues communicate under healthy and pathological conditions through the products they secrete [15,16,17]. This communication may occur within the same organ or between distant organs through intracrine, paracrine, and endocrine mechanisms. Therefore, considering the common features shared by PCOS and MetS, it is understandable that there is intimate communication between the liver and adipose tissue in both conditions [16,18] (Figure 1).

The liver performs both endocrine and exocrine functions to communicate with other tissues [19,20,21]. Approximately 60% of liver parenchymal cells are hepatocytes, whereas 30–35% are non-parenchymal cells, such as Kupffer cells (KC), hepatic stellate cells (HSCs), liver sinusoidal endothelial cells, and resident hepatic macrophages [22]. Hepatocytes account for nearly 80% of the liver’s volume and have a high capacity for regeneration after injury; however, they rarely divide under normal conditions. Hepatic cells express and release several proteins into the bloodstream, including proteins related to innate immunoglobulins and complement factors [20]. Many acute-phase proteins secreted by the liver are regulated by proinflammatory cytokines, such as tumor necrosis factor-alpha (TNF-α) and interleukin-6 (IL-6) [21,22,23,24].

Beyond their role in innate immunity, hepatocytes perform a central metabolic function in regulating glucose and lipid homeostasis [25,26,27,28]. Hepatocytes are the first cells to come into contact with material absorbed from the gastrointestinal tract. Thus, they participate in protein and lipid metabolism and in the removal of apoptotic cells from circulation. Although hepatocytes contribute to the development of liver fibrosis under abnormal conditions, hepatic stellate cells (HSCs) are the principal drivers of fibrosis progression [23,27,28]. Systemic glucose homeostasis is regulated by the liver through glucose production and glycogen storage. When necessary, the liver increases glucose output to supply non-hepatic tissues. The liver also supplies lipids to peripheral tissues via very-low-density lipoprotein cholesterol (VLDL-C) production to deliver triglycerides as energy substrate. Communication between the liver and other tissues, including adipose tissue, occurs in part through hepatokines, which transmit information about hepatic metabolic status (Table 1) [23]. Some hepatokines have beneficial effects while others negatively affect adipose tissue metabolism.Additionally, some cytokines have a dual effect, depending on blood concentration or clinical conditions.

Adipocytes play a central role in lipid uptake and storage [29,30]. They store excess energy as triglycerides, but because their storage capacity is limited, lipid overflow may lead to ectopic fat deposition in other organs, which occurs in PCOS [21,31]. In addition to storing excess fat, the adipocytes produce various hormones, cytokines, and free fatty acids (FFAs). When secreted by visceral adipocytes, these substances drain directly into the portal vein, reach hepatic circulation, and affect both the parenchymal cells (hepatocytes) and immune cells (KCs) [27,31]. Hepatic fat accumulation of more than 5%, driven primarily by portal FFA influx, is a key contributor to chronic metabolic disorders such as insulin resistance and T2DM, which are implicated in the development of cardiovascular disease (CVD) [32,33]. Lipids, peptides, and proteins, such as TNF-α, as well as several other cytokines released by adipocytes, reach hepatocytes and exert multiple effects [15,33,34,35,36] (Table 2).Therefore, the accumulation of visceral adipose tissue, particularly hypertrophic adipocytes, in the omental and mesenteric regions is a major marker of metabolic dysfunction, largely due to the excessive delivery of FFAs to the liver [33,37]. Furthermore, as previously mentioned, the proinflammatory cytokines produced by hypertrophic visceral adipocytes directly target hepatic cells via the portal circulation [37,38,39,40,41].

The close relationship between adipocytes and hepatocytes reflects a common developmental origin. Beyond energy storage functions, adipocytes have immuno–metabolic activities, including mobilizing FFAs and communicating with distant tissues through adipokine secretion [40,41]. The main adipokines and their assigned roles are listed in Table 2 [42]. Central obesity in PCOS, also a major component of MetS, contributes to a variety of metabolic and inflammatory disturbances [8,9]. The impact of hypertrophic adipocyte-derived products on liver function is of considerable scientific interest because increased lipid flux to the liver promotes hepatic triglyceride accumulation [13]. Notably, the prevalence of obesity in women with PCOS exceeds 50%, compared with about 35% in women without [3,43]. Visceral adiposity produces most adipokines that contribute to liver injury [44].

## 3. Liver and Metabolic Dysfunction in Women with PCOS

**Figure 2:**
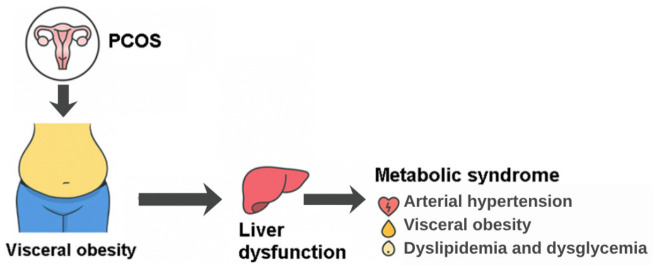
Liver metabolic dysfunction in obese women with PCOS.

The association between PCOS, particularly the obese phenotype, and liver dysfunction is supported by both clinical and laboratory evidence [45,46,47]. Considering that obesity, a component of MetS, is present in 50–70% of women with PCOS, an excess of adipose tissue contributes to hepatotoxicity, which leads to liver dysfunction in both PCOS and MetS [2,39,48,49] (Figure 2). As already mentioned, in obese PCOS, the large adipose tissue mass delivers high amounts of FFAs to the liver, where they are esterified into triglycerides within hepatocytes [50]. When microvesicular triglyceride accumulation exceeds 5% of hepatocytes, women with PCOS are at risk of developing several metabolic disorders, including hepatic hypoxia, chronic inflammation, steatosis, fibrosis, and MASLD [50,51,52]. Excessive hepatic fat accumulation also impairs insulin sensitivity [51] and acts as a local proinflammatory stimulus, activating liver-resident macrophages (KCs) [53,54,55]. Consequently, hepatic lipotoxicity induces oxidative stress (OS) and chronic inflammation, contributing to the development of MetS, MASLD, and metabolic dysfunction-associated steatohepatitis (MASH) in PCOS [53,56].

Oxidative stress in the liver promotes the overproduction of reactive oxygen species (ROS), such as homocysteine, malondialdehyde, asymmetric dimethylarginine, hydrogen peroxid (H_2_O_2_), and the hydroxyl radical [56,57]. In PCOS, the protective antioxidant mechanisms are impaired and correlated with hyperinsulinemia, hypertension, and dysglycemia [25,55]. Furthermore, reactive oxygen species (ROS) activate various protein kinases that phosphorylate serine/threonine residues of insulin receptor substrate-1 (IRS-1), resulting in IR, hepatic inflammation, hepatocellular damage, and lipid peroxidation [57,58,59].

## 4. Adipocyte and Metabolic Dysfunction in Women with PCOS

**Figure 3:**
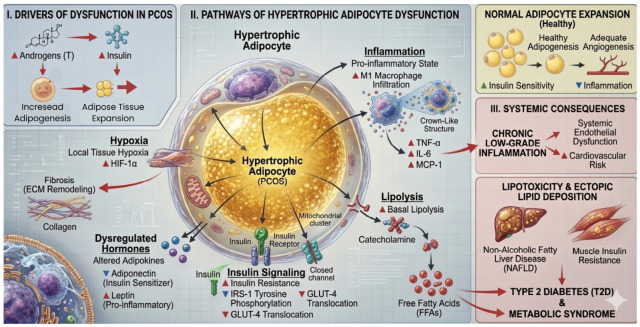
Hypertrophic adipocyte dysfunction in women with PCOS.

In PCOS, androgens have receptors in both preadipocytes and mature adipocytes [60]. PCOS hyperandrogenism is linked to increased visceral fat accumulation [61], which is accompanied by upregulated transcription of chemokine (C-C motif) ligand 2 (CCL2). This upregulation triggers macrophage infiltration [62] and increases the production of proinflammatory cytokines [61,63,64] (Figure 3). In hyperandrogenic PCOS phenotypes, most visceral adipocytes are hypertrophic and associated with the oversecretion of potentially harmful adipokines, such as leptin, visfatin, and TNF-α, in addition to reduced secretion of beneficial adipokines (e.g.,adiponectin and omentin), resulting in dyslipidemia, dysglycemia, and IR [64,65]. In women with PCOS, the enlarged adipocytes are also present in the subcutaneous adipose tissue [66].

Overall, adipose tissue dysfunction in PCOS is strongly linked to metabolic complications [21,67]. An imbalance in adipokines, characterized by higher leptin levels and lower levels of adiponectin, as seen in hyperandrogenism, positively correlates with the body mass index (BMI), waist-to-hip ratio (WHR), and abnormalities in carbohydrate and lipid metabolism [66,68,69,70].Various adipokines have receptors on hepatocytes [67,68,69], and their specific effects on the liver are mediated through canonical or noncanonical signaling pathways [59,71].

## 5. The Role of Hepatokines in Metabolic Dysfunction in Women with PCOS

**Figure 4:**
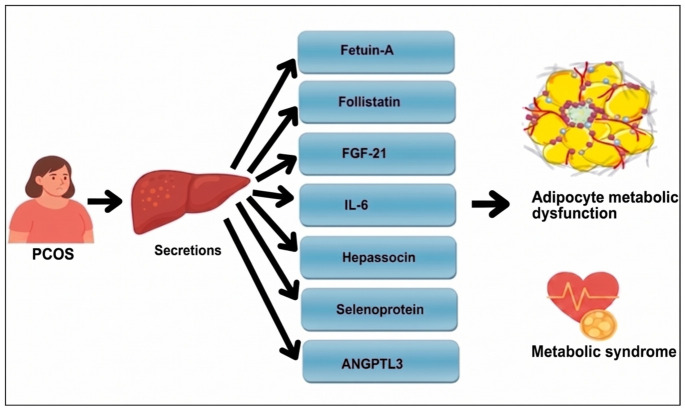
The role of hepatokines on adipocytes’ metabolic dysfunction in women with PCOS.

Various secreted hepatokines locally influence the liver’s metabolic status in addition to acting on distant tissues [21,72,73]. As previously mentioned, hepatokines may exert beneficial, harmful, or dual metabolic effects [21,72]. This section focuses on selected hepatokines with beneficial, detrimental, or both effects in women with obesity and PCOS (Figure 4).

### 5.1. Hepatokines with Beneficial or Dual Effects

Fibroblast growth factor 21 (FGF-21) is predominantly secreted by the liver, and its levels reflect the degree of fat hepatic accumulation [74]. At physiological levels, FGF-21 acts on the liver by decreasing oxidative stress, improving insulin sensitivity, reducing steatosis, and regulating glucose metabolism. In normal adipocytes, FGF-21 suppresses lipolysis, limits triglyceride synthesis, and promotes fatty acid oxidation. Thus, FGF-21 may exert beneficial actions. By activating the adenosine monophosphate-activated protein kinase (AMPK) and histone protein diacetylasesirtuin 1 (Sirt 1) pathways, FGF-21 increases mitochondrial oxidative capacity, reduces oxidative stress [75], and decreases lipid biosynthesis, while promoting fatty acid β-oxidation, improving hepatic insulin sensitivity, reducing VLDL-C influx, and attenuating endoplasmic reticulum stress [76]. Therefore, it may ameliorate some hepatic disorders [77,78,79,80]. In white adipocytes, FGF-21 increases insulin-independent glucose uptake by increasing the expression of glucose transporter type 1 (GLUT1), suppressing lipolysis, and promoting fatty acidoxidation. Conversely, when chronically elevated in some conditions, such hyperglycemic and hyperandrogenic states, it disrupts carbohydrate and lipid metabolism in the liver [81,82]. In women with PCOS, FGF-21 levels are elevated, independent of BMI, and are associated with increased FFA, insulin levels in circulation, inflammation, IR, liver injury, and the development of MASLD [81,83]. By unclear mechanisms, under certain conditions FGF-21 suppresses adiponectin secretion, induces lipolysis in adipocytes, and increases FFA release [84,85,86].

Follistatin, which is primarily secreted by the liver [87,88,89], under normal conditions, and locally, it attenuates fibrosis and steatosis, increases glucose uptake, and promotes liver regeneration. In adipocytes, follistatin promotes differentiation, prevents hypertrophy, and improves insulin sensitivity. Also, it stimulates irisin secretion and inhibits activin production. In abnormal conditions, such as PCOS, follistatin levels are increased [90], likely due to genetic polymorphism. When increased, it antagonizes transforming growth factor beta (TGFβ) ligands, which may lead to oxidative stress. Independent of obesity, follistatin positively correlates with fasting insulin, oral glucose tolerance test (OGTT) results [91], pancreatic B-cell function, insulin sensitivity regulation, and low-grade chronic inflammation [92,93,94]. Additionally, it correlates positively with C-reactive protein (CRP), the free androgen index (FAI) [94], induces IR, increases lipolysis, promotes FFA release [93], correlates with total cholesterol (TC), low-density lipoprotein cholesterol (LDL-C), TG, homeostatic model assessment for insulin resistance (HOMA-IR), and negatively correlateswith HDL-C [95]. In non-PCOS subjects, it may also impair glucose uptake under certain conditions [95,96]. Due to its dual effect, it may have therapeutic potential in improving insulin sensitivity. Although its applicability is limited by the suppression of activin-A and FSH, worsening of anovulation, and increasedandrostenedione [95].

Interleukin-6 (IL-6), which is expressed by adipocytes, hepatocytes, HSCs, and fibroblasts [97,98], has hepatoprotective roles, including cytoprotection, regeneration, and certain metabolic benefits [98,99,100,101,102]. Other interleukins, including the IL-20 subfamily (IL-19, IL-20, IL-22, IL-24, and IL-26), mediate communication between leukocytes and epithelial cells, promoting anti-apoptotic and anti-fibrotic effects in the liver [103]. IL-22 may improve insulin sensitivity, glucose tolerance, and inflammation in PCOS [104]. IL-6 induces an acute-phase response, increasing hepatic CRP and VLDL-C production under certain conditions [99,100]. Its expression also promotes hepatic IR in non-human models [104]. In women with PCOS, particularly in the liver, IL-6 promotes fibrosis, insulin resistance, and inflammation. IL-4 and IL-13 activate KCs (macrophages), increasing the secretion of inflammatory mediators and promoting lipid accumulation in hepatocytes [102,103,104]. Additionally, interleukins suppress the expression of the adiponectin and visfatin gene, and contribute to lipid mobilization when acutely increased. In contrast, chronic elevation of interleukins may exert anti-inflammatory effects on macrophages but proinflammatory effects on T cells [103,104].

### 5.2. Hepatokines with Harmful Effects

Hepassocin activates extracellular regulated kinases 1 and 2 (ERK1 and ERK2) in adipocytes, reducing adipogenesis and improving insulin sensitivity. Alternatively, in the liver, high levels of hepassocin promote fat accumulation, impair glucose metabolism, and promote TG accumulation, lipogenesis, liver injury, and inflammation, contributing to the development of MASLD [105,106]. In PCOS, hepatossocin shows a positive correlation with HOMA-IR, LDC-C, and total testosterone levels, and a negative correlation with BMI and waist circumference (WC) [105]. The functions of high levels of hepassocin on adipose tissue are still unclear, but animal and cell model studies suggest that it worsens IR and promotes fat accumulation [106]. Hepassocin has gained recent attention as a potential link between liver health, PCOS, and MetS [106]. It serves as a non-invasive analyte for assessing early metabolic hepatic involvement before substantial changes appear on an elastography. As limitations, its levels may vary with specific PCOS phenotypes. Additionally, there is no reference range for hepassocin levels, and there is no specificity, while it is linked to PCOS and MetS Furthermore, hepassocin is also elevated in other conditions such as T2DM and liver injury. Finally, most clinical data come from small, cross-sectional studies rather than long-term longitudinal trials. Currently, it is not possible to affirm that lowering hepassocin levels directly improves PCOS features.

Selenoprotein-P (Sep-P) is a hepatokine involved in glucose metabolism. Its circulating levels are increased in PCOS, and correlate positively with testosterone and HOMA-IR, and negatively with WC and HDL-C [107,108]. Sep-P is also associated with oxidative stress-mediated liver damage and fibrosis severity in women with PCOS [72]. It has received recent attention as a potential link between liver health, PCOS, and MetS.

Angiopoietin-like protein-3 (ANGPTL3), which is exclusively secreted by hepatocytes, inhibits lipoprotein lipase (LPL) by promoting its dissociation into inactive monomers, thereby regulating plasma TGs [109,110]. Particularly in the liver, ANGPTL3 inhibits LPL and endothelial lipase, regulating lipid metabolism. Additionally, it increases hepatic uptake of fructose-derived metabolites and regulates triglyceride-rich lipoprotein secretion [109]. In adipocytes, ANGPTL3 interaction with LPL promotes TG storage in the white adipose tissue [110,111], disrupts carbohydrate and lipid homeostasis, and modulates VLDL-C metabolism in an endothelial lipase-dependent manner [112]. ANGPTL-3 also induces lipolysis and suppresses lipogenesis [113]. Its circulating levels are increased in obesity and positively correlated with plasma levels of glucose, insulin, HOMA-IR score, and MASLD [114,115]. Low ANGPTL3 levels are associated with decreased TG, LDL-C, and HDL-C [116]; its levels are higher in PCOS compared with controls [117], and are positively correlated with BMI and TG, and negatively correlated with HDL-C levels. Overall, ANGPTL3 contributes to dyslipidemia and IR in PCOS.

Circulating levels of the hepatokine fetuin-A are increased in overweight and obese women with or without PCOS, due to upregulation by the influx of FFAs [118,119,120]. Fetuin-A is associated with the development of IR by inhibiting insulin receptor tyrosine kinase and activating Toll-like receptor signaling, thereby impairing glucose uptake [121,122]. Fetuin-A also activates the inflammatory pathways and oxidative stress, contributing to the development of metabolic disorders, and facilitating the progression of MASLD to metabolic dysfunction-associated steatohepatitis (MASH) [123,124,125]. Clinically, fetuin-A is positively associated with BMI, TGs, TC, HOMA-IR, and glycated hemoglobin (HbA1C), and negatively associated with LDL-C levels in women with and without PCOS [126,127,128]. Additionally, independent of adiposity, circulant fetuin-A correlates with liver fat content, early atherosclerosis, and MetS [128,129], and activates the extracellular signal-regulated-kinase (ERK½) and nuclear factor-kappa B (NF-kB) pathways [130]. In adipocytes, fetuin-A inhibits insulin receptor phosphorylation, promotes IR, diminishes adiponectin production, and induces adipocyte hypertrophy [121].

Fetuin-B, another hepatokine, is also a hepatocyte-derived factor involved in glucose metabolism [131,132]. Its circulating levels are increased in women with PCOS and, in the liver, are positively associated with IR, TG accumulation, steatosis, and the development of MASLD. Fetuin-B’s mechanisms of action are not yet fully understood, but it appears to affect glucose metabolism by inhibiting cysteine proteases [133,134]. Additionally, it decreases AMPK activity and downregulates fatty acid oxidation, although it does not induce proinflammatory signaling. In adipocytes, it inhibits insulin-mediated glucose uptake. In women with PCOS, fetuin-B is associated with a high risk of CVD [134,135].

## 6. The Role of Adipokines in Metabolic Dysfunction in PCOS

**Figure 5:**
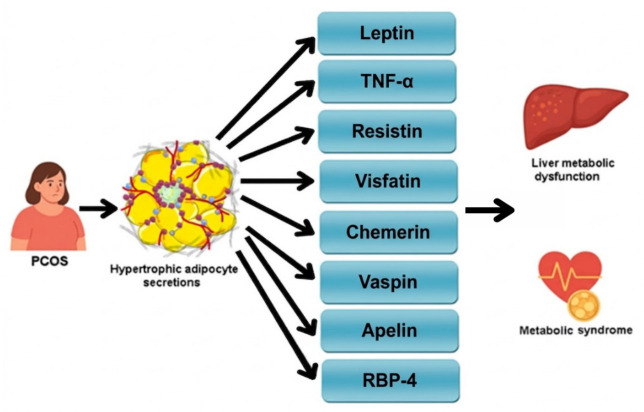
The role of adipokines inliver metabolic dysfunction in women with PCOS.

Many adipokines affect liver and pancreatic β-cell functions in women with PCOS, resulting in inflammation, gluconeogenesis, reactive oxygen species (ROS) production, dyslipidemia, liver steatosis, apoptosis, and fibrosis (Figure 5). Several adipokines also act on adipocytes themselves via paracrine and autocrine mechanisms.

### 6.1. Adipokines with Beneficial or Dual Effects

Under physiological conditions, leptin exerts beneficial effects on hepatic glucose and lipid metabolism [136]. It reduces lipid accumulation, promotes lipid mobilization, prevents steatosis [137,138], and improves insulin resistance [138,139]. Additionally, in the liver, leptin stimulates fatty acid oxidation [138]. Under pathological conditions, such as PCOS and obesity, leptin levels are increased and exert detrimental effects, being positively correlated with adiposity, insulin, and testosterone levels. Moreover, leptin polymorphisms are associated with elevated alanine aminotransferase (ALT), fatty liver [140,141], increased expression of TGF-β1 in endothelial and Kupffer cells, profibrotic effects, and inhibition of HSC apoptosis [142]. Leptin also correlates with the severity of MASLD and hepatic IR [141]. Its fibrogenic effect is modulated by the sympathetic nervous system via norepinephrine-mediated HSC activation [142]. Leptin activates lipid metabolism and stimulates KCs to release inflammatory cytokines [139].Further, leptin also suppresses glucose-induced insulin secretion in pancreatic β cells [143], inhibits hepatic phospholipase activity, and enhances glycogen storage in hepatocytes [144,145,146]. In addition, adipocytes promote lipolysis [26,147] and counteract the effects of insulin [145].

Adiponectin, which is produced by adipocytes, is a beneficial cytokine with an important hepatic effect. Its receptor 2 (AdipoR2) is highly expressed in hepatocytes [148], where it acts via AMPK and peroxisome proliferator-activated receptor alpha (PPARα) pathways to increase FFA oxidation, decrease gluconeogenesis, reduce FFAs influx, and de novo lipogenesis, thereby limiting hepatic fat accumulation [149,150]. Adiponectin exerts anti-inflammatory and antifibrotic effects on hepatocytes and KCs. In hepatic sinusoidal cells, it suppresses inflammatory cytokines and induces anti-inflammatory cytokines [151]. It also prevents hepatocyte apoptosis [151]. Its antifibrotic activity involves inhibiting HSC activation and proliferation via downregulating TGF-B1, and increasing extracellular matrix degradation. Adiponectin counteracts hepatic lipid storage, either in normal or high levels [43,152,153]. The levels of adiponectin are decreased in obesity, T2DM, and systemic arterial hypertension [154]. In subjects with these conditions, hypoadiponectinemia is associated with liver disease, especially with MASLD, IR, dyslipidemia, and CVD [155]. Hypoadiponectinemia predicts hepatic steatosis, ALT elevation, and increased gamma–glutamyl transferase (GGT) [155]. In PCOS, adiponectin levels are low and associated with MetS [151,155,156], reduced insulin sensitivity, and dyslipidemia. In adipocytes, it enhances insulin-mediated glucose uptake and reduces the expression of proinflammatory adipokines [157,158].

Chemerin is produced as an inactive precursor (prochemerin) in visceral adipose tissue and the liver. It is rapidly converted via proteolysis and subsequently secreted in an active form [159,160]. In normal conditions, chemerin presents anti-inflammatory effects acting on adipogenesislipid, and glucose metabolism [161,162]. When its levels are increased, such obesity, T2DM, PCOS, and MetS, it is positively associated with BMI, fasting glucose, TG, blood pressure, TNFα, and IL-6 levels [163,164]. Chemerin levels are associated with the expression of KCs and TNFα [163], and may not reflect the hepatic expression or hepatic effect on steatosis pathogenesis. However, elevated levels of chemerin have been associated with liver fibrosis and portal inflammation [163,164,165]. In humans, increased chemerin levels are associated with MASLD [160]. Chemerin pathogenic effects are related to obesity, hyperinsulinism, and low-grade chronic inflammation [166,167,168]. Additionally, in adipocytes, it increases lipogenesis, decreases glucose uptake, and attracts macrophages.

Omentin, a cytokine expressed in visceral adipose tissue (VAT), is also found in various tissues [169], in which it exerts an anti-inflammatory effect. In the liver, it exerts anti-inflammatory effects, decreases apoptosis, and attenuates oxidative stress [169]. In adipocytes, it enhances insulin-mediated glucose uptake and reduces the expression of proinflammatory adipokines [170,171]. Omentin circulant levels are inversely related to IR, T2DM, obesity, and MetS [170,171,172,173]. In women with PCOS presenting with IR, the levels of omentin are lower [173] and negatively associated with CVD [173].

Vaspin is expressed in adipose tissue, in the liver, and in some endocrine cells. Usually, vaspin acts as an anti-inflammatory cytokine with the capacity to reduce oxidative stress and increase insulin sensitivity through inhibiting proteases that degrade insulin signaling [174,175]. In adipocytes, vaspin reduces hypertrophy, decreases production of proinflammatory cytokines, and increases insulin signaling. However, its circulating levels may be higher in obesity and T2DM [176], and increased levels in the liver are associated with the development of MASLD and MASH, but it does not appear to be associated with hepatic steatosis and inflammation [175,177]. Although a few studies have associated vaspin with hepatic fibrosis [178,179], recently it was shown that vaspin, in fact, can reduce hepatic fibrosis by increasing AMPK activity and downregulating NF-kB expression [177]. Considering that vaspin expression is enhanced in granulosa cells in obese women and stimulates steroidogenesis in ovarian follicles [174], some studies have reported on the role of vaspin in PCOS, a condition associated with obesity [177]. Clinically, vaspin is correlated with anthropometric parameters, TG levels, and insulin resistance in T2DM patients [178,179,180,181]. Intriguingly, in PCOS, vaspin levels have been found to be decreased [178], increased [180], or even unchanged [181]. The conflicting findings of vaspin in women with PCOS across studies may be due to different phenotypes, insulin levels, populations, or study design. The first correlation of vaspin with PCOS reported higher levels of vaspin in this syndrome, probably due tovaspin resistance [181], without correlating to BMI or HOMA-IR score [180]. Polymorphism in the vaspin gene may be associated with risk for MetS [182]. Most research is restricted to animal models or observational human studies. Well-designed studies in humans are needed to clarify when the effects of vaspin are beneficial or harmful.

Apelin, which is expressed in the liver, adipose tissue, and granulosa cells [183], is involved in glucose and lipid metabolism [184,185]. In adipocytes, it inhibits adipogenesis and lipolysis, increases glucose uptake via AMPK, and shows antioxidant effects. In the liver, however, apelin acts as a profibrotic factor via HSCs and promotes angiogenesis and cirrhosis, but protects against lipid accumulation. Its levels are elevated in obesity, insulin resistance, hepatic cirrhosis, and MASLD [186]. In hepatic steatosis, apelin circulating levels are normal. Its receptors are predominantly expressed in obese PCOS women [182] and are positively correlated with BMI, menstrual cycle duration, and HOMA-IR score, and negatively correlated with follicle-stimulating hormone (FSH) levels [186].A correlation between apelin and IR in women with PCOS is uncertain [185,187], but apelin was shown to play a role in follicular growth arrest in the pathophysiology of polycystic ovary morphology (PCOM) by inhibiting follicular development and increasing insulin expression [183]. Insulin stimulates apelin secretion, and higher levels of apelin can be found in MetS [188,189,190]. Despite some positive correlations with BMI, HOMA-IR, fasting glucose, TG, and free testosterone levels, and a negative correlation with HDL-C [191,192,193], more studies are needed on the contradictory role of apelin in PCOS.

### 6.2. Adipokines with Harmful Effects

Resistin is highly expressed in hypertrophic adipocytes [194]. In women with PCOS, resistin levels may be increased, although a few studies have reported unchanged levels [195,196,197,198]. Resistin is produced by macrophages infiltrating adipose tissue, peripheral blood mononuclear cells, and hepatic HSCs [199,200]. It acts as a proinflammatory cytokine by stimulating other inflammatory factors (TNF-α, IL-Lβ, IL-6, and IL-12) in macrophages and mononuclear cells; by itself, it can also be induced by inflammatory mediators [201]. In the liver, and when elevated, resistin promotes inflammation and moderate fibrosis by activating HSCs, increasing the production of TGF-β and type 1 collagen by KCs [201,202,203]. Resistin is frequently increased in obesity, PCOS, and T2DM, counteracting insulin’s effect, favoring gluconeogenesis, increasing glucose output, and inhibiting AMPK in hepatocytes [198]. The increased levels of resistin in obesity are associated with systemic arterial hypertension (SAH) [199,200], liver fat accumulation, and type 2 diabetes mellitus (T2DM) [198,199,200,201,202]. In PCOS, its higher circulating levels, independently of obesity, are associated with visceral obesity and increased androgen levels [198,199,203]. Finally, in adipocytes, resistin increases lipid content during cell maturation and stimulates other proinflammatory cytokines [203,204].

Visfatin is produced in various tissues. In adipose tissue, visfatin is produced by macrophages infiltrating the adipocytes [205]. In adipocytes, visfatin inhibits macrophage apoptosis, thereby prolonging their activity and secretion [206]; additionally, through its proinflammatory action, visfatin promotes the production of other proinflammatory cytokines [206]. At a distance, visfatin affects pancreatic β-cell activity by inhibiting nicotinamide phosphoribosyl transferase (NAMPT) [207,208]. For the most part, in the liver, visfatin induces oxidative stress, apoptosis, glucose and lipid metabolism abnormalities, IR, and inflammation, promoting liver adiposity and the expression of fibrotic biomarkers, [209,210,211]. Levels of visfatin are increased in women with PCOS, in whom itexerts effects on IR, hyperandrogenism, and metabolic dysfunction, and induces proinflammatory markers [212]. Retinol binding protein-4 (RBP-4) is expressed and secreted in the liver and visceral adipocytes. It participates in the transport of vitamin A from the liver to peripheral tissues [213,214]. In the liver, RBP-4 is related to liver fat accumulation [215] and the induction of the gluconeogenesis enzyme phosphoenolpyruvate carboxykinase (PEPCK) expression, resulting in increased glucose levels [216]. RBP-4 concentrations are elevated in insulin resistance, obesity, dypolycystic ovary syndrome; metabolic syndrome; adipokines; hepatokines; hepatic steatosis; hyperandrogenismlipidemia, and high blood pressure states [217,218]. Higher levels have also been associated with MASLD [219], liver steatosis [220,221], and fibrosis [222,223]. Overall, higher RBP-4 levels are associated with an increased risk of metabolic diseases, such as T2DM and MASLD [224]. It is also associated with TG accumulation, leading to hepatic mitochondrial dysfunction through decreased oxidative capacity [224,225,226,227]. In adipocytes, RBP-4 activates the macrophage production of proinflammatory cytokines, decreases insulin signaling in both lean and obese individuals, and increases lipolysis. In PCOS, circulating levels of RBP-4 are increased even after adjusting for age and BMI [228], particularly in hyperandrogenic phenotypes [229]. In this syndrome, RBP-4 is positively correlated with HOMA-IR and hyperglycemia, and negatively correlated with fasting glucose levels. In PCOS, the pathogenesis and role of RBP-4 in IR are at odds since higher levels have been reported in some [229,230], but not all [231,232,233,234], studies. Concerning androgen levels, the current findings are also conflicting, as studies report either no association [228,235] or positive association [229,236]. Currently, the utility of RBP-4 in PCOS and MetS remains inconclusive and a subject of active debate [220]. RBP-4 early rise before clinical metabolic changes can be identified and may be a warning sign and an independent predictor of CVD. However, the RDP-4 role is limited by a lack of assay specificity, and some conflicting findings regard carbohydrate metabolism. The lack of a normal range for RBP-4 concentrations also limits its use in clinical practice. Its best use is a part of assessing long-term metabolic and cardiovascular risk [233].

Tumor necrosis factor alpha (TNF-α) is a proinflammatory cytokine secreted by macrophages, granulosa cells, adipocytes, monocytes, lymphocytes, natural killer (NK) cells, and hepatocytes [237]. Through autocrine mechanisms, in the liver, TNF-α promotes hepatocyte proliferation, which plays a crucial role in hepatic regeneration [238]. However, it inhibits insulin signal transmission, affects glucose metabolism, promotes fat accumulation, and exerts proinflammatory effects. Therefore, by its action on the liver, TNF-α is involved in the development of metabolic diseases, such as obesity, PCOS, IR, and T2DM. In adipocytes, it stimulates lipolysis, inhibits adipogenesis, and induces insulin resistance. TNF-α levels are elevated in PCOS [237,238,239,240] independent of obesity, and linked to IR, hyperandrogenism, and systemic inflammation [237,240]. In addition, hypertriglyceridemia and hyperinsulinemia in PCOS trigger TNF-α secretion and fatty liver accumulation [236,241,242].

## 7. The Role of Adipokines/Hepatokines in the Development of Metabolic Syndrome in Women with PCOS

PCOS shares several factors with MetS, including dysglycemia, dyslipidemia, hyperinsulinemia, and elevated blood pressure. Specifically, decreased HDL-C, elevated TG levels, increased WHR, and IR are highly prevalent in both PCOS and MetS. Additionally, both conditions are strongly associated with CVD [9]. PCOS is also associated with the expansion of VAT and increased secretion of adipokines [243]. Most adipokines and hepatokines are elevated in both PCOS and MetS [244,245,246]. However, adiponectin and omentin levels are lower [247]. Elevated adipokine levels are implicated in the development of MetS in women with PCOS [248]. Indeed, depending on the population studied, over 30% to 50% of women with PCOS meet the criteria for MetS diagnosis [249].

The liver is the main site of glycogen storage and insulin clearance and regulation, making it a key organ in the development of MetS. Increased visceral fat in PCOS clearly contributes to MetS pathogenesis, as FFAs reach the liver via portal circulation.The metabolic influence of cytokines has been extensively studied, as in the present study. As noted in this review, the specific mechanisms through which cytokines, when present at either increased or decreased levels, contribute to MetS remain incompletely understood and are largely based on indirect evidence. However, elevated androgen levels may affect adipokine production [249] by activating macrophage-derived proinflammatory cytokines in adipose tissue [246,250]. Overall, visceral adiposity accompanied by dysglycemia, dyslipidemia, chronic inflammation, and hypertension in PCOS contributes to the development of MetS [249].

### 7.1. Effect of Adipokines on Coexistence of Polycystic Ovary Syndrome and Metabolic Syndrome

Given that leptin regulates VAT expansion, in cases of leptin resistance, adipose tissue accumulation persists with increased leptin production, which is accompanied by decreased adiponectin, a profile marker of MetS [251,252]. As stated previously, leptin is implicated in inflammation, oxidative stress, arterial hypertension, atherosclerosis, and IR [253,254]. It has been reported to facilitate the development of MetS [251,252]. Further, in PCOS, leptin exacerbates IR and hyperandrogenism, partially by inhibiting aromatase [252]. There is an extensive body of literature on the occurrence of hyperleptinemia in PCOS and MetS. However, studies on leptin levels specifically in women with the association of comorbid PCOS and MetS are limited [255,256]. Finally, comparisons between women with PCOS with and without MetS indicate that anthropometric and glycolipid parameters are worse when the two conditions coexist [256]. Even though leptin levels appear to be even higher in the combined PCOS and MetS, the intrinsic mechanism remains unclear [254,257]. In summary, higher levels of leptin are implicated in reproductive and metabolic health. In PCOS, it moves from being a helpful factor to a driver of abnormalities. It bridges an unbalanced LH/FSH ratio, preventing ovulation, inhibiting ovarian steroidogenesis and follicle development, and correlating with adiposity. Because the current models of investigation fail to explain why lean and obese women with PCOS suffer from similar dysfunctions, it remains unclear whether hyperleptinemia causes metabolic dysfunction or IR is what drivesabnormalities. Since leptin drivesup blood pressure by increasing the sympathetic nervous system, it is considered a robust biomarker to predict future development of T2DM, MetS, and cardiovascular events. Because of the existence of leptin resistance, no current leptin-target drug is available.

Resistin polymorphisms have also been observed in the coexistence of MetS and PCOS, particularly in hyperandrogenic PCOS [258,259,260]. Higher resistin levels in PCOS appear to be related to MetS development. Although results are somewhat inconsistent [258,259,260], in hyperandrogenism, the elevated levels of resistin increase hepatic glucose production and promote arterial hypertension [261,262,263]. Despite resistin being described as a predictor of MetS in PCOS, and various studies have described the role of resistin in MetS in different populations, reports on the coexistence of PCOS and MetS are scarce. In short, resistinbridges adipose tissue, inflammation, and IR. In PCOS, it contributes to metabolic and reproductive dysfunctions, since it enhances theca cell androgen production, whilesimultaneously promoting IR. RegardingMetS, resistin leads to vascular damage by upregulation of adhesion molecules and other proinflammatory cytokines. Currently, resistin is not recommended for routine screening or clinical diagnosis of PCOS and MetS.

In PCOS, as previously reported, visfatin maybe associated with obesity, T2DM, MetS, and CVD [264,265]. Higher visfatin levels in women with MetS [266] are associated with fasting plasma glucose, HbA1C, insulin resistance, HOMA-IR score, HDL-C, TG, and many predictors of MetS [266]. Elevated visfatin levels in women with or without PCOS have also been shown to correlate with low HDL-C, high BMI, and the FAI [266]. Despite the similarity between PCOS and MetS, data on visfatin in the combination of MetS and PCOS are limited, although elevated levels in both conditions may suggest worsening clinical presentation. As primary strengths, visfatin can bind to the insulin receptor at a differentsite, helping to understand why glucose uptake occurs, even in IR states [267]. In PCOS and MetS, the higher visfatin levels are linked to endothelial dysfunction and subclinicalatherosclerosis. Some factors limit the use of visfatin in clinical practice: it can beelevated in various inflammatory conditions, and there are no standardized assays for its measurement. Further, it remains unsolved whether high visfatin levels cause dysmetabolism or if this alteration is a consequence of increased adipose tissue.

Chemerin appears to be a promising biomarker of MetS as it mediates systemic proinflammatory activity and IR [268,269,270,271]. Certain chemerin polymorphisms appear to increase the risk of MetS [272,273]. As previously described, increased chemerin in PCOS is independent of BMI and obesity, and this increase is associated with ovarian hyperandrogenism, obesity, IR, dyslipidemia, chronic inflammation, and dysglycemia [274]. In both obese and nonobese PCOS populations, higher levels of chemerin are correlated with the WHR. In PCOS and MetS, chemerin is a marker of disease severity and driver of underlying pathology [268]. In PCOS, elevated chemerin levels inhibitfolliculogenesis and interferewith testosterone production, contributing to infertility [166]. Regarding the mechanisms of chemerin effects on PCOS and MetS, they remain debatable. It is not clear whether chemerincauses or is simply a marker of the expanded adipose tissue. In a clinical setting, the measurement of chemerin for PCOS diagnosis is limited because its assays have no specificity and measure several active and inactive isoforms as total chemerin. Moreover, there are no cut-off or reference range values established across ages and ethnicity [166]. Studies assessing chemerin levels in women with PCOS associated with MetS are very scarce, and they have suggested worsening of metabolic parameters [275].

Elevated levels of TNF-α have been found in patients with bothPCOS and MetS [276,277]. It is important to highlight that TNF-α impairs insulin function in the liver, which is associated with hyperandrogenism, insulin resistance, and obesity, common features of both PCOS and MetS. In women with PCOS, TNF-α polymorphism appears to increase patient susceptibility to Mets [278], due to its detrimental role in carbohydrate and lipid metabolism and increased ROS accumulation [279,280]. Considering the common features of PCOS and MetS, it is plausible to hypothesize that their combination may amplify the risks of CVD in these conditions [281]. However, the role of TNF-α has not been fully explored in women with PCOS who also present with MetS [282], but the proinflammatory and ROS-related effects shared by both conditions may contribute to severe long-term metabolic consequences [283,284]. The primary strength of studying TNF-α is its well-defined effect on PCOS and MetS. In addition to its metabolic role, TNF-α stimulates theca cells to produce testosterone, which promotes visceral fat accumulation.Moreover, TNF-α secretion creates a self-perpetuating feedback. Despite its clear mechanistic role, the use of TNF-α in a clinical setting has significant obstacles [284]. Its measurement lacks specificity, and it is unclear if the higher levels of TNF-α are a result of PCOS or a consequence of obesity. Regarding TNF-α measurement, significant heterogeneity among studieshas been demonstrated [284]. Although anti-TNF-α therapies have shown some benefits in animal models for reducing androgen levels and inhibiting weight gain, there are no benefits for the clinical management of PCOS and MetS [284].

### 7.2. The Effect of Hepatokines on the Coexistence of Polycystic Ovary Syndrome and Metabolic Syndrome

Hepatokines are released from the liver in response to stress, hormones (androgens insulin, glucagon, and glucocorticoids), and metabolic or nutritional states [22]. They are implicated in the development of PCOS and MetS through their regulation of carbohydrate, lipid, and protein metabolism [285] (Figure 3).

As mentioned before, IL-6 levels rise in certain abnormal conditions and can promote insulin resistance, facilitating the development of MetS and CVD [286,287,288,289,290]. IL-6 levels are increased in PCOS and MetS, particularly in obese individuals [289,291]. Because MetS and PCOS share most pathophysiological factors, such as obesity, dyslipidemia, dysglycemia, and hypertension [124,292], it is reasonable to speculate an increased risk of CVD in the combination of both conditions, especially due to chronic inflammation and exacerbated oxidative stress [293]. As a strength IL-6, in both PCOS and MetS, is the most reliable indicator for chronic subclinical inflammation and CVD risk in PCOS. By inducing expression of the suppressor of cytokine signaling 3 (SOCS3), IL-6 impairs insulin signaling in the ovarian follicle and promotes development of esteroidogenesis and hyperandrogenism in PCOS [294,295]. However, its practical clinical use is limited. Its measurement has a few limitations. It lacks specificity as a diagnostic tool for PCOS because it presents high variability during the day, sleep, stress, and meals. Further, because the IL-6 molecule is derived from muscle, which improves glucose uptake and has an anti-inflammatory effect, it has no potential therapeutic value due to compensation by other cytokines.As far as we can ascertain, no study has examined IL-6 concentrations specifically in women with PCOS associated with MetS.

Fetuin-A, which is present at higher levels in both PCOS and MetS, blocks insulin receptor function and reduces adiponectin secretion by adipocytes, increasing liver fat accumulation [296,297]. As mentioned earlier, higher levels of fetuin-A in PCOS correlate positively with fasting glucose, fasting insulin, HOMA-IR, TC, TG, and LDL-C, indicating a strong correlation with metabolic dysfunctions [76]. In women with MetS, fetuin-A levels are also elevated [298,299,300] and are related to a large WC, increased LDL-C, decreased HDL-C, dysglycemia, and hypertension [127,301,302]. Even though increased fetuin-A levels are even more pronounced in the association between PCOS and MetS, the effects of the combination of both syndromes on metabolism are not clear [303]. This highlights the need for further research to better understand the interaction between fetuin- A, PCOS, and MetS. In clinical practice, fetuin-A has a potential diagnostic role, since it represents an easy analyte to screen for IR. However, it has not been used in routine clinical procedures because some inconsistencies regarding its levels in PCOS and MetS have been observed, possibly dependent on PCOS phenotype, the existence of high levels in other dysmetabolic diseases, and the lack of a standardized cut-off. Mostly, the current knowledge is based simply on observational cohort or case-control studies [124,126,296].

As noted earlier, selenoprotein-P (Sep-P) is a hepatokine observed at increased levels in fatty liver, PCOS, and T2DM [304]. In women with PCOS, Sep-P levels are inversely correlated with fasting glucose, insulin, and HOMA-IR score, and positively correlated with the LDL-C, hypertriglyceridemia, WHR, and testosterone levels [304,305]. Additionally, Sep-P is inversely correlated with adiponectin, confirming impaired glycolipid metabolism [306]. Sep-P is also associated with hepatic steatosis and fibrosis [304,307], even in individuals without PCOS. However, some studies do not support an association between Sep-P and MetS [308]. These inconsistent results are also observed in women with PCOS [309,310]. The role of Sep-P in women with both MetS and PCOS has not been adequately evaluated. An experimental study showed that the administration of purified Sep-P impairs insulin signaling and glucose metabolism in hepatocytes, indicating possible use in a therapeutic scenario [108]. However, despite the association of higher levels of Sep-P with PCOS-related comorbidities [305], the underlying mechanisms remain unclear and require further investigation.

In humans, FGF-21 is associated with obesity-related metabolic diseases, likely due to FGF-21 resistance [284,311,312]. FGF-21 levels are positively correlated with lipids and arterial hypertension. In a clinical setting, FGF-21 has been accepted as a highly sensitive and specific biomarker for PCOS diagnosis, being a useful and strong indicator of liver steatosis and hepatic and systemic metabolic imbalance [313,314], although its short half-life and the existence of resistance limit its use [315]. Despite some cases showing FGF-21 resistance and the short half-life, it may have a potential therapeutic role in metabolic diseases. FGF-21 administration in rodents and non-human primateshas demonstrated benefits in obesity-related dysmetabolic conditions [316]. In women with T2DM and MASH, FGF-21 administration of a stable analogue demonstrated to be effective on dyslipidemia, hepatic fat, and serum markers of liver fibrosis [316]. However, it did not improve dysglycemia [316]. In women with PCOS, FGF-21 levels are associated with other metabolic disturbances [81,83,312,313]. FGF-21 levels are also higher in MetS [81,83,315,316]. However, FGF-21 has not been specifically evaluated in women with PCOS associated with MetS.

As aforementioned, ANGPTL3 is implicated in lipid and carbohydrate metabolism, and its levels have been reported to be increased in T2DM and MetS [317,318]. In women with PCOS, ANGPTL3 levels are positively correlated with higher BMI, androgens, HOMA-IR score, and fasting glucose, and negatively correlated with HDL-C levels [117,319]. However, this hepatokine has not been evaluated in association with PCOS and MetS. The relevance of ANGPTL-3 in PCOS and MetS stems from its regulation of TG and association with IR and MASLD [318]. Therefore, in the context of MetS, this is a regulation of atherogenic lipids [319]. Due to its role in raising TG, ANGPTL3 inhibitor vupanorsen is a potential drug for therapeutic use [320]. Currently, studies using antisense nucleotides to reduce ANGPTL3 have demonstrated a significant reduction in TG, VLDL-C, FFA, and insulin resistance in adipose tissue [320]. Additionally, it improves IR and lowers ovarian hyperandrogenism in PCOS. It must be highlighted, however, that, currently, it lacks large-scale data confirming the use of ANGPTL3 inhibitors to reduce hirsutism and restore ovulation.

Leukocyte cell-derived chemotoxin-2 (LECT2) [321,322] is a hepatokine linked to hepatic inflammation, hepatic adiposity, and IR [322]. It has a positive correlation with BMI, WC, HOMA-IR, T2DM, and liver fat deposition [322,323]. Its levels are increased in individuals with visceral obesity and abnormal lipid metabolism [324,325]. This hepatokine has not been sufficiently investigated in women with PCOS, and its association with MetS remains uncertain. Some studies suggest elevated levels in dyslipidemia and MetS [326,327], but further research is needed.

## 8. Study Limitations

The understanding of PCOS has advanced significantly with the identification of the complex crosstalk between adipose tissue- and liver-derived factors. However, a critical analysis of the current literature reveals several methodological and conceptual gaps limiting the immediate clinical application of these findings. Some limitations should be considered: First, a narrative review provides a thoughtful, readable, and practical synthesis of a topic; however, unlike a systematic review, it does not involve a formal quality assessment of the included studies. This limitation is particularly relevant in areas with conflicting evidence, such as the divergent data regarding circulating levels of vaspin and FGF-21, precluding their use in clinical practice as both diagnostic and therapeutic targets for PCOS to counteract metabolic abnormalities. Second, considering the current knowledge, it is difficult to determine if dysregulated hepatokines and adipokines are intrinsic features of PCOS or secondary to visceral adiposity. Androgen excess suggests an intrinsic role as it favors android obesity fatty liver and inflammation. Third, heterogeneity among studies, such as the inclusion of different PCOS phenotypes as a single group, may explain distinct cytokine profiles. Furthermore, variations in ethnicity, age, and dietary habits make it unlikely that a universal biomarker for PCOS and MetS can be established. Fourth, most of the human clinical data discussed in this review are derived from observational studies; while these studies identify associations between specific cytokines and metabolic dysfunctions, they do not establish causality. Moreover, it remains unclear whether altered hepatokine levels precede the onset of MetS or represent a secondary response to pre-existing hepatic lipotoxicity. Current research suggests that while certain hepatokines are secondary to liver stress, others act as primary drivers predicting MetS. Fifth, a limited number of studies have specifically evaluated women presenting with both PCOS and MetS simultaneously. Consequently, most of the current evidence is extrapolated from studies assessing these conditions separately. Additionally, several hepatokines discussed in this review, such as LECT2 and ANGPTL3, remain poorly investigated in the context of PCOS or MetS. Sixth, much of the mechanistic understanding of how cytokines activate inflammatory pathways or impair insulin receptor signaling is derived from in vitro experiments or animal models. These findings may not be perfectly translated to humans, in whom the interplay of sex steroids and insulin creates a much more complex metabolic environment. Well-designed longitudinal and mechanistic studies in humans are therefore necessary to clarify the association of hepatokines and adipokines in the metabolic abnormalities found in both PCOS and MetS.

## 9. Concluding Remarks

Cytokines are only partially responsible for communication between the liver and adipocytes. The beneficial, harmful, or dual effects of certain hepatokines and adipokines on adipose tissue and liver are complex, and their capacity to improve or worsen metabolic parameters in women with PCOS or other abnormal conditions is regulated through an autocrine, paracrine, and endocrine network. The evidence presented in this review emphasizes that PCOS extends far beyond reproductive disorder, manifesting as a complex metabolic condition driven by hyperandrogenism and dysfunctional adipose tissue–liver crosstalk. The dysregulation of specialized signaling protein molecules, adipokines, and hepatokines acts as a primary driver for the development of insulin resistance, dyslipidemia low-grade chronic inflammation, and MetS in women with PCOS. A central mechanism linking these two organs is the influx of FFAs and adipokines from visceral adipose tissue to the liver, triggering a cascade of lipotoxicity and oxidative stress. In addition to local effects, the altered secretion of hepatokines may further modulate adipocyte secretion and exacerbate systemic metabolic failure. Moreover, the reduction of the protective adipokines, such as adiponectin and omentin, can accelerate hepatic dysfunction.

Regarding specific hepatokines in PCOS, the higher levels of fetuin-A in the liver result from FFA influxand are associated with obesity, IR, inflammation, and oxidative stress. In adipocytes, fetuin-A inhibits insulin receptor phosphorylation, aggravates IR, blocks adipogenesis, and promotes low-grade chronic inflammation. In a clinical setting, fetuin-A is a useful analyte to screen for insulin resistance in PCOS, but it has not yet been used in routine procedures due to inconsistencies observed in its levels in PCOS and MetS. Increased FGF-21 levels in PCOS reflect the degree of hepatic steatosis and are associated with liver injury, IR, and inflammation. In adipocytes, it enhances insulin signaling, regulates lipolysis, and promotes fatty acid oxidation. Although some cases have shown FGF-21 resistance, it may have a potential therapeutic role, since its administration in rodents and non-human primates demonstrated some benefits in obesity-related dysmetabolic conditions. Further, in humans, administration of a stable analogue has been demonstrated to be effective on dyslipidemia, hepatic steatosis, and hepatic fibrosis. IL-6 in the liver increases CRP and VLDL-C production, and promotes IR, although it also exerts cytoprotective and regenerative effects. In adipocytes, normal levels of IL-6 may enhance insulin sensitivity, increase glucose uptake, and improve lipid profile under physiological conditions. However, its chronic activation and higher levels, as observed in PCOS, by shifting macrophages from the anti-inflammatory M2 phenotype to proinflammatory M1 phenotype, promote chronic inflammation, IR, fibrosis, and steatosis, and impair adipocyte metabolism. IL-6 is the most reliable indicator of subclinical chronic inflammation in PCOS, but its measurement is limited by a lack of specificity and outcome variability during the day, stress, meals, and sleep.

In normal conditions, resitin decreases fibrosis, attenuates insulin action, and increases glucose output. In PCOS, it moves from being a useful factor to a driver of abnormalities. In the liver, resistin promotes inflammation, fibrosis, and the synthesis of type 1 collagen and TGF-B. In adipocytes, resistin promotes inflammation, insulin resistance, and reduces glucose uptake. In PCOS, it enhances ovarian androgen production, favoring IR and MetS. It was shown that resistin leads to vascular damage. However, it is not recommended for routine screening of PCOS and MetS. Increased levels of leptin in the liver are associated with steatosis, fibrosis, and IR. In adipocytes, leptin promotes lipolysis, counteracts insulin action, and facilitateshyperandrogenism. It remains unclear why lean and obese women with PCOS can present similar dysfunctions. Leptin is considered a good biomarker for predicting the development of MetS and cardiovascular events in women with or without PCOS. In the liver RBP4 promotes steatosis and fibrosis, and induces gluconeogenesis and IR. In adipocytes, RBP4 increases lipolysis, stimulates macrophages to release proinflammatory adipokines, and reduces GLUT-4 activity. In the liver, TNF-α promotes hepatocyte proliferation and hepatocyte regeneration but inhibits insulin signal transmission, resulting in dysglycemia and T2DM. In adipocytes, TNF-α increases lipolysis, induces apoptosis, and increases IR, resulting in dyslipidemia. The use of TNF-α in a clinical setting is limited by assays without specificity, and there are heterogeneous results reported between studies. Despite some benefits of anti-TNF-α use in animal models, there are no recommendations regarding its use in the management of PCOS and MetS. Finally, some adipokines, such as chemerin, omentin-1, and vaspin, may exert beneficial metabolic effects depending on the physiological context.

Taken together, these findings suggest that dysregulated crosstalk between adipose tissue and the liver represents a central mechanism linking PCOS and MetS. Understanding this complex network of hepatokines and adipokines may improve the identification of women at higher risk of CVD and support the development of targeted therapeutic strategies. Despite its limitations, this review provides a comprehensive framework for future research, highlighting the need for well-designed studies to further elucidate these mechanisms.

## Figures and Tables

**Table 1:** The functional role of hepatokines in adipose tissue.

**Metabolic Benefit**	**Actions**
FGF21	Decreases fatty mass Decreases oxidative stress Decreases triglycerides Decreases apoptosis Increases insulinsensitivity Decreases steatosis
MANF	Decreases inflammation Decreases lipolysis Increases energy expenditure Insulin resistance
ORM	Decreases adipogenesis Decreases fibrosis adipose tissue
Activin	Decreases thermogenesis Regulates glucose metabolism Improves insulin sensitivity
Follistatin	Increases glucose uptake Decreases insulin sensitivity
**Metabolic Harm**	**Actions**
ANGPTL3	Promotes hyperglycemia Promotes hyperlipidemia Increases insulin resistance Increases release FFAs
Fetuin-A	Increases inflammation Blocks glucose uptake Increases insulin resistance
Follistatin (high levels)	Positive correlation with insulin, TC, LDL-C, TG, HOMA-IR Negative correlation with HDL-C
FGF-21 (high levels)	Promotes liver injury Induces insulin resistance Promotes liver inflammation
GPNMB	Favors fat accumulation Increases expression lipogenic genes Increases insulin resistance

Adapted fromY Zhang et al., *Genes and Diseases*, 10:825, 2023 [23]; Abbreviations: MANF = mesencephalic astrocyte-derived neurotrophic factor; ORM = orosomucoid; ANGPTL3 = angiopoietin-like proteins; FFA = free fatty acid; WAT = white adipose tissue; GPNMB = glycoprotein nonmetastatic melanoma protein B. FGF21 = fibroblast growth factor 21; TC = total cholesterol; LDL-C = low-density lipoprotein cholesterol; TG = triglycerides; HOMA-IR= homeostatic model assessment IR; HDL-C = high-density lipoprotein cholesterol.

**Table 2:** The functional role of adipokines in the liver.

**Metabolic Benefits**	**Actions**
Omentin	Reduces oxidative stress Reduces apoptosis Increases glucose uptake
Apelin	Decreases steatosis Decreases oxidative stress
Adiponectin	Decreases hepatic fibrosis Anti-inflammatory effect Decreases cell damage Reduces TNF-α production Promotes fatty acid oxidation Increases insulin sensitivity
Chemerin	Decreases oxidative stress Anti-inflammatory effect
Leptin	Reduces lipid accumulation Prevents steatosis Promotes lipid metabolization
Vaspin	Suppresses apoptosis Increases insulin sensitivity Decreases oxidative stress Anti-inflammatory effects
**Metabolic Harm**	**Actions**
Visfatin	Proinflammatory activity Promotes insulin resistance Increases apoptosis Promotes steatosis Promotes fibrosis
TNF-α	Promotes hepatic fibrosis Favors lipid peroxidation Promotes apoptosis Promotes necrosis
Leptin (high levels)	Promotes hepatic fibrosis Increases oxidative stress Promotes steatosis Promotes lipolysis Inhibits insulin action
Resistin	Increases lipid accumulation Increases proinflammatory cytokines
RBP-4	Increases lipogenesis Promotes steatosis Induces proinflammatory effect
IL-6	Promotes hepatic fibrosis Promotes inflammatory effect Suppresses gene adiponectin Suppresses gene visfatin

Adapted from: K Zhao et al., *Hepatology Communications*. 9: e0639, 2025 [42]. Abbreviations: TNF-α = tumor necrosis factor alpha, IL-6 = interleukin-6, RBP-4 = retinol-binding protein.

## Data Availability

There search data are not publicly available on legal or ethical grounds. In addition, all data produced and analyzed during this study were included in this article. Further inquiries can be directed to the corresponding author.
